# Syndromic surveillance: STL for modeling, visualizing, and monitoring disease counts

**DOI:** 10.1186/1472-6947-9-21

**Published:** 2009-04-21

**Authors:** Ryan P Hafen, David E Anderson, William S Cleveland, Ross Maciejewski, David S Ebert, Ahmad Abusalah, Mohamed Yakout, Mourad Ouzzani, Shaun J Grannis

**Affiliations:** 1 Department of Statistics, Purdue University, West Lafayette, Indiana, USA; 2 Department of Mathematics, Xavier University, New Orleans, Louisiana, USA; 3 Department of Electrical and Computer Engineering, Purdue University, West Lafayette, Indiana, USA; 4 Department of Computer Science, Purdue University, West Lafayette, Indiana, USA; 5 Regenstrief Institute, Indianapolis, Indiana, USA; 6 School of Medicine, Indiana University, Indianapolis, Indiana, USA

## Abstract

**Background:**

Public health surveillance is the monitoring of data to detect and quantify unusual health events. Monitoring pre-diagnostic data, such as emergency department (ED) patient chief complaints, enables rapid detection of disease outbreaks. There are many sources of variation in such data; statistical methods need to accurately model them as a basis for timely and accurate disease outbreak methods.

**Methods:**

Our new methods for modeling daily chief complaint counts are based on a seasonal-trend decomposition procedure based on loess (STL) and were developed using data from the 76 EDs of the Indiana surveillance program from 2004 to 2008. Square root counts are decomposed into inter-annual, yearly-seasonal, day-of-the-week, and random-error components. Using this decomposition method, we develop a new synoptic-scale (days to weeks) outbreak detection method and carry out a simulation study to compare detection performance to four well-known methods for nine outbreak scenarios.

**Result:**

The components of the STL decomposition reveal insights into the variability of the Indiana ED data. Day-of-the-week components tend to peak Sunday or Monday, fall steadily to a minimum Thursday or Friday, and then rise to the peak. Yearly-seasonal components show seasonal influenza, some with bimodal peaks.

Some inter-annual components increase slightly due to increasing patient populations. A new outbreak detection method based on the decomposition modeling performs well with 90 days or more of data. Control limits were set empirically so that all methods had a specificity of 97%. STL had the largest sensitivity in all nine outbreak scenarios. The STL method also exhibited a well-behaved false positive rate when run on the data with no outbreaks injected.

**Conclusion:**

The STL decomposition method for chief complaint counts leads to a rapid and accurate detection method for disease outbreaks, and requires only 90 days of historical data to be put into operation. The visualization tools that accompany the decomposition and outbreak methods provide much insight into patterns in the data, which is useful for surveillance operations.

## Background

The development of statistical methods for accurately modeling disease count time series for the early detection of bioterrorism and pandemic events is a very active area of research in syndromic surveillance [1-15]. Early detection requires accurate modeling of the data.

A challenge to modeling is systematic components of time variation whose patterns have a level of predictability – inter-annual (long-term trend), day-of-the-week, and yearly-seasonal components [2,10]. The variation can be ascribed to causal factors: for the inter-annual component the factor can be an increase or decrease in the population of people from which patients come; for the yearly-seasonal component it is changing weather and human activities over the course of a year; and for the day-of-the-week component it is a changing propensity of patients to seek medical attention according to the day of the week. In addition to these components is a noise component: random errors not assignable to causal factors that often can be modeled as independent random variables.

The most effective approach to early outbreak detection is to account for the systematic components of variation and the noise component through a model, and then base a detection method on values of the systematic components and the statistical properties of the random errors. Methods that do not accurately model the components can suffer in performance. The model predicts the systematic recurring behavior of the systematic components so it can be discounted by the detection method, and specifies the statistical properties of the counts through the stochastic modeling of the noise component. The two dangers are lack of fit – patterns in the systematic components are missed – and overfitting – the fitted values are unnecessarily noisy. Both are harmful. Lack of fit results in increased prediction squared bias, and overfitting results in increased prediction variance. The prediction mean-squared error is the sum of squared bias and variance.

Many modeling methods require large amounts of historical data. Examples are the extended baseline methods [11-15] of the Early Aberration Reporting System (EARS) [4], which require at least 3 years of data. Another popular example is a seasonal autoregressive integrated moving average (ARIMA) model in [3] that is fitted to 8 years of data. Such methods are not useful for many surveillance systems that have recently come online, so methods have been proposed that require little historical data [16]. They typically employ moving averages of recent data, such as C1-MILD (C1), C2-MEDIUM (C2), and C3-ULTRA (C3), which are used in EARS. But such methods do not provide optimal performance because they do not exploit the full information in the data; they smooth out some component effects and operate locally to avoid others, rather than accounting for the components.

Our research goal is accurate modeling of components of variation, but without requiring a large amount of historical data. The research was carried as part of our analysis of the daily counts of chief complaints from emergency departments (EDs) of the Indiana Public Health Emergency Surveillance System (PHESS) [17]. The complaints are divided into eight classifications by CoCo [18]. Data for the first EDs go back to November 2004, and new EDs have come online continually since then. There are now 76 EDs in the system.

There are many ways to proceed in modeling chief-complaint time series. One is to develop parametric models. However, our new modeling methods are based on a nonparametric method, seasonal-trend decomposition using loess (STL) [19], which is very flexible and can account for a much wider range of component patterns than any single parametric model. Just as importantly, it can be used with as little as 90 days of data. We have also developed a new synoptic-scale (days to weeks) outbreak detection method based on the STL modeling.

## Methods

### Data

The STL decomposition was run on all Indiana EDs for respiratory and gastro-intestinal counts. Both are fundamental markers for a number of naturally occurring diseases, and research has shown that diseases from bioweapons have early characterization of influenza-like illness [20], which typically results in respiratory complaints.

We present results for the respiratory time series for the 30 EDs that came online the earliest; these series end in April 2008, and start at times ranging from November 2004 to September 2005. All analyses were performed using the R statistical software environment [21], and an R package is available as a supplemental download [see additional file 1].

### Modeling

STL decomposes a time series into components of variation [19] using a local-regression modeling approach, loess [22]. Our methods use STL to decompose the square root of ED daily counts into four components:(1)

where *t *is time in units of years and increments daily, *Y*_*t *_is the respiratory count on day *t*, *T*_*t *_is an inter-annual component that models long-term trend, *S*_*t *_is a yearly-seasonal component, *D*_*t *_is a day-of-the-week component, and *N*_*t *_is a random-error noise component.

**Figure 1:**
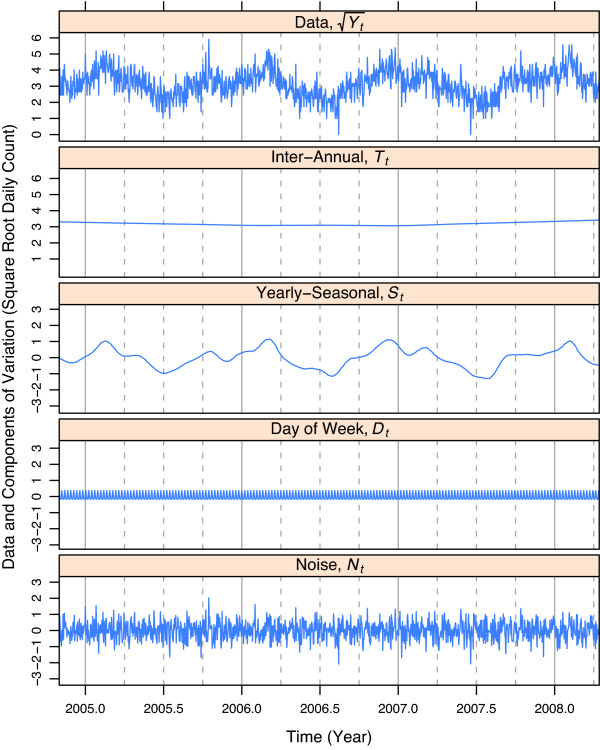
**STL decomposition for respiratory square root daily counts**. Respiratory square root daily counts and four components of variation of the STL decomposition for an Indiana emergency department (ED). The four components sum to the square root counts. The solid vertical lines show January 1 and the dashed vertical lines show April 1, July 1, and October 1.

Figure 1 shows the STL decomposition of  for one ED. *T*_*t *_is nearly constant. *S*_*t *_has peaks due to seasonal influenza: single peaks in early 2005, 2007, and 2008, and a double peak in late 2006 and early 2007. While some of these yearly-seasonal effects are visible in the raw data, the effects are much more effectively seen in *S*_*t *_because other effects including the noise are not present. Variation in *D*_*t *_is small compared with the total variation of , but it cannot be ignored in the modeling because its variation is not small compared with the initial growth of critical disease outbreaks. *N*_*t *_is irregular in behavior, and can be accurately modeled as independent, identically distributed normal random variables with mean 0. The square root transformation results in much simpler statistical properties. For example, the standard deviations of the *N*_*t *_are nearly constant within and between hospitals, and the marginal distribution is nearly normal with mean 0 and variance . Neither are true for the untransformed counts. The behavior of the *N*_*t *_after transformation is not surprising because the raw counts can be thought of as Poisson random variables, and the square root of a Poisson random variable whose mean is not too small is approximately normal with a standard deviation of 0.5 [23].

STL components of variation arise from smoothing the data using moving weighted-least-squares polynomial fitting with a moving window bandwidth in days. The degree of the polynomial is 0 (locally constant), 1 (locally linear), or 2 (locally quadratic).

The inter-annual component, *T*_*t*_, arises from locally linear fitting with a bandwidth of 1000 days, a very low-frequency component. (Window bandwidths can be formally larger than the number of available data points.)

For the yearly-seasonal component, *S*_*t*_, a critical idea of our method is not pooling values across years with the same time of year – values of January 1, values of January 2, etc. – as is often done. STL methodology can provide pooling, but because our methods are designed to work with limited data, *S*_*t *_at a time *t *uses data in a neighborhood of *t*, and not across years. *S*_*t *_is a low-frequency component with a bandwidth of 90 days and with a blending of locally quadratic and locally constant fitting. It is possible that this local method would be better in many surveillance applications even with substantially more data if the phase and shape are sufficiently variable from one year to the next. For example, for some Indiana EDs, seasonal influenza peaks are unimodal in some years and bimodal in others. Our modeling tracks this accurately, but averaging across years could easily distort the patterns, for example, making the bimodal peaks merge into unimodal ones. Other research has also found that the "one season fits all" assumption, leading to pooling across years, is not suitable for disease surveillance data [10].

The blending for the *S*_*t *_results in nearly stationary noise, *N*_*t*_. Local smoothing methods tend to produce systematic components that have higher standard deviation as the time approaches the ends of the data, making the noise component have a lower standard deviation at the ends. We countered this with a new smoothing method, blending. *S*_*t *_results from blending locally quadratic smoothing and locally constant smoothing, a weighted average of the two for the 50 days at each end of the data. The weight for the quadratic smoother is 0.7 at the first or last observation, and increases linearly moving away from each end, becoming 1 at the 50th observation from each end. This means the weight for the constant smoother goes from 0.3 to 0.

The fitting algorithm begins with computation of a day-of-the-week component, *D*_*t*_, directly employing the method described in [19]. STL can allow *D*_*t *_to slowly evolve though time but we found that *D*_*t *_is a strictly periodic component. *D*_*t *_results from an iterative process. First, a low-middle-frequency component is fitted using locally linear fitting with a bandwidth of 39 days. Then *D*_*t *_is the result of means for each day-of-the-week of the  minus the low-middle-frequency component. Then the current *D*_*t *_is subtracted from the  and the low-middle-frequency component is re-computed. This iterative process is continued to convergence. We subtract the final day-of-the-week component from the , and then use loess smoothing on the result to obtain the inter-annual component *T*_*t *_as described above. Finally, we subtract the day-of-the-week and inter-annual components from the  and smooth the result to obtain *S*_*t *_as described above.

We selected the stable *D*_*t *_assumption and the bandwidths of *T*_*t *_and *S*_*t *_through extensive use of visualization and numerical methods of model checking in order to prevent overfitting or a lack of fit of the components of variation, and to keep two components from competing for the same variation in the data.

### Outbreak Detection

STL modeling provides public health officials with a clear view of yearly-seasonal variation for visual monitoring of the onset and magnitude of seasonal peaks. This is facilitated by the smoothness of *S*_*t *_as opposed to the raw data.

Another critical task is the detection of outbreaks that rise up on the order of several days to several weeks and are not part of the systematic patterns in disease counts; the causes are bioterrorism and uncommon highly pernicious diseases. Following the terminology of meteorology, we will refer to this as a "synoptic time-scale outbreak".

Our synoptic-scale detection method uses a simple control chart based on the assumption that the daily counts are independently distributed Poisson random variables with a changing mean *λ*_*t*_. These assumptions are validated in the results section. The variability in the systematic components *T*_*t*_, *S*_*t*_, and *D*_*t *_is taken to be negligible which is reasonable since they are relatively low-frequency components. We take all variability to be in *N*_*t*_. From Equation 1,(2)

 is estimated by the sample standard deviation of the *N*_*t*_.

With *Y*_*t *_as a Poisson random variable with with mean *λ*_*t*_, our method declares an outbreak alarm when *Y*_*t *_results in a value *y*_*t *_for which *P *(*Y*_*t *_≥ *y*_*t*_; *λ*_*t*_) is less than a threshold *ρ*. We evaluate this synoptic-scale outbreak method using the historical data for baselines and adding artificial outbreaks, as has been done in other research [24,25]. We use three baselines: respiratory daily counts from three EDs with low, medium, and high daily means – 9.25, 17.33, and 23.86.

**Figure 2:**
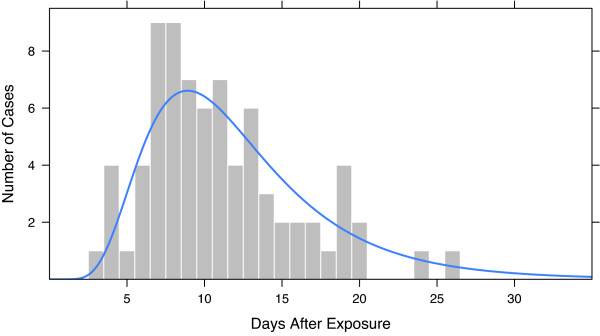
**Outbreak sample**. Randomly generated outbreak of 76 cases injected according to the Sartwell model [26] is shown by the histogram. The lognormal density of the model multiplied by 76 is shown by the curve. The parameters of the lognormal are the mean, *ζ *= 2.401, and the standard deviation, *σ *= 0.4626, both on the natural log scale.

The outbreak model is a lognormal epicurve of Sartwell [26]. A starting day is selected and becomes day 1. A total number of cases, *O*, is selected; the bigger the value of *O*, the more readily the outbreak can be detected. The day of each case is a random draw, rounded to the nearest positive integer, from a lognormal distribution whose minimum is 0.5. For our evaluation, with the lognormal density as(3)

we use *ζ *= 2.401 and *σ *= 0.4626 to approximate temporal behavior of an anthrax outbreak [27]. The lognormal density times a constant is shown in Figure 2, which will be fully explained later.

Detectability at a point in time depends on the the number of outbreak cases per day compared with the variability of the baseline counts over the outbreak period. As we have emphasized earlier, the variability of an ED count time series is larger when the level of the series is larger than when the level is smaller. Because *O *is fixed, detectability changes through time.

We used three values of *O *for each baseline, selected based on a method in [1]. There are many ways to make the selection, but we chose this one to remain consistent with past work. The maximum of our lognormal epicurve is 0.087 and occurs at  = 8.9 days. If we suppose the density is this value for 8.9 ± 0.5 days, then the expected number of cases in this peak interval is 0.087*O*. Detectability can be controlled by making the expected peak-interval cases equal to a magnitude factor *f *times the sample standard deviation of the residual counts *Y*_*t *_- (*T*_*t *_+ *S*_*t *_+ *D*_*t*_)^2^. The parameter *f *controls the detectability.

The three values of *O *in our evaluation for each baseline were chosen by this method with *f *= 1, 1.5, and 2. For example, for the baseline with the smallest mean daily counts, the standard deviation of the residual counts is 3.324. For *f *= 2, we have 2 × 3.324/0.087 = 76.414, and rounding to the nearest integer, *O *= 76. This case is shown in Figure 2. The curve is the lognormal density times 76, and the histogram shows 76 draws from the lognormal.

We compared this synoptic-scale method to other methods. Because some of our surveillance EDs have a rather small number of observations, we compared to the EARS C1, C2, and C3 methods [4]. These methods are based on a simple control chart approach with the daily test statistic calculated as(4)

where *μ*_*t *_and *σ*_*t *_are a 7-day baseline mean and standard deviation. For the C1 method, the baseline window for *μ*_*t *_and *σ*_*t *_is days *t *- 7,..., *t *- 1 and for the C2 method, the baseline window is days *t *- 9,..., *t *- 3. The test statistic for the C3 method is *S*_*t *_+ *S*_*t*-1 _+ *S*_*t*-2_, where *S*_*t*-1 _and *S*_*t*-2 _are added if they do not exceed the threshold.

Since some EDs have more than 3 years of data, we also considered some of the EARS longer historical baseline methods. However, we chose not to implement any of them since it has been shown that these do not offer much of an advantage over the limited baseline methods [16]. Instead, we compared with a Poisson generalized linear model (GLM) method [24], which has terms for day-of-the-week, month, linear trend, and holidays. We did not include holidays since we were not able to find the details of its implementation. We also compared with a seasonal ARIMA model [3] but found its results unsatisfactory, so we do not include it here. This could be due to insufficient data for pooling across years, or that such pooling through the seasonal terms of the model cannot accommodate the substantial change from one year to the next in the seasonal patterns.

Outbreak detection was tested using 1004 days of each baseline. The first 365 days acted as historical data for the GLM method to have sufficient data to estimate coefficients. For each of the 9 combinations of outbreak detectability and baseline, we simulated 625 different outbreaks; the first started on day 366, the second on day 367, and so forth to the final on day 990.

Each outbreak was a different random sample from the lognormal epicurve. One sample is shown in Figure 2. The counts of each outbreak were added to the baseline counts to form *Y*_*t*_. We ran each method through each day of an outbreak as would be done in real practice, and tracked whether the outbreak was detected and, if so, how long it took to detect. Time until detection is measured by the number of days after first exposure until the day of detection. Any evaluation that did not result in detection on or before day 14 of the outbreak was classified as not-detected.

We also investigated the minimum amount of data needed by the STL method to achieve good performance. Because *T*_*t *_is very stable, and the window for *S*_*t *_is 90 days, we would expect STL to do well for 90 days or more. To test this, we ran our methods at each time point from 366 to 990 using just the most recent 90 days of baseline data for each outbreak scenario.

We ran all methods at a false positive rate of 0.03, achieved empirically by choosing a cutoff, *ρ*, for each method from the historical data with no outbreaks injected. For the C1, C2, and C3 methods, this means choosing *ρ *to be the 97th percentile of the daily test statistic values. Since the STL and GLM methods update past fitted values as they progress, choosing *ρ *for these methods cannot be done by simply fitting the entire time series and retrospectively choosing *ρ*. Instead, we use the fitted values obtained at the last day of each fit over time to chose *ρ*.

In practice, limits for the STL and GLM methods would be set by using the theoretical false positive rate. If a model fits the data, the observed rates should be consistent with the theoretical rate. We compared the GLM and STL methods by studying their observed rates for the 30 EDs using *ρ *= 0.03.

## Results

### STL Modeling

**Figure 3:**
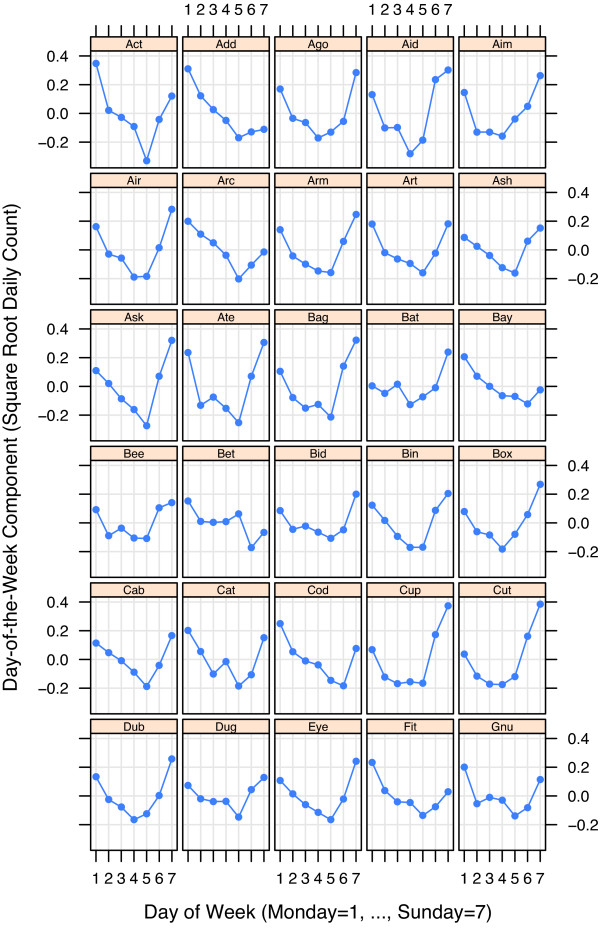
**Day-of-the-week component**. Day-of-the-week component, *D*_*t*_, for square root respiratory counts for 30 Indiana EDs. The general pattern is U-shaped with a Monday or Sunday maximum and a Thursday or Friday minimum.

**Figure 4:**
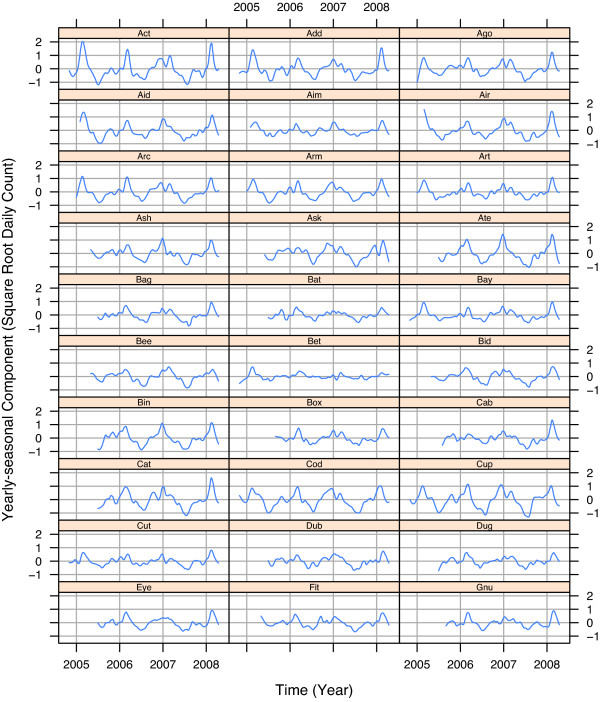
**Yearly-seasonal component**. Yearly-seasonal component, *S*_*t*_, for square root respiratory daily counts for 30 Indiana EDs. Overall, patterns are similar, but detailed behavior varies with unimodal and bimodal peaks, different times of onset of yearly-seasonal disease, and different times of disease peaks.

**Figure 5:**
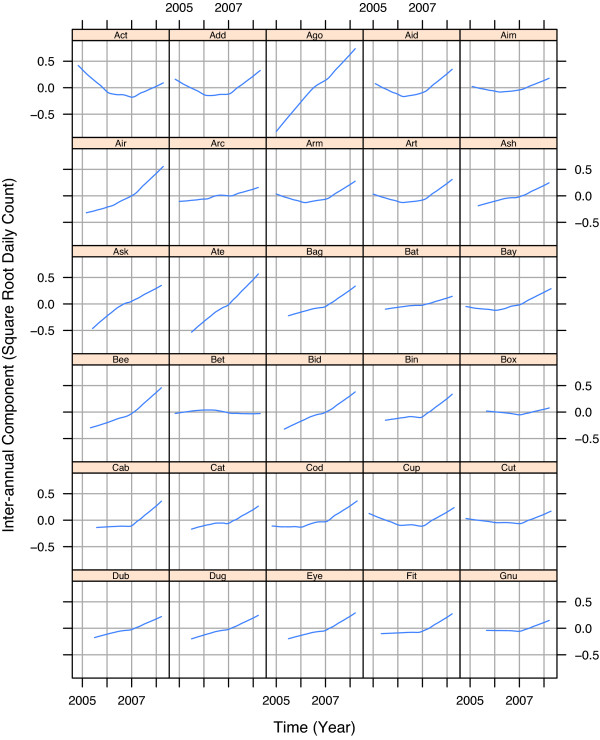
**Inter-annual component**. Inter-annual component, *T*_*t*_, for respiratory square root counts for 30 Indiana EDs. Each component has been centered around zero to protect anonymity. The long-term trend for each ED is either nearly constant or has a small increase due to a growing patient population.

**Figure 6:**
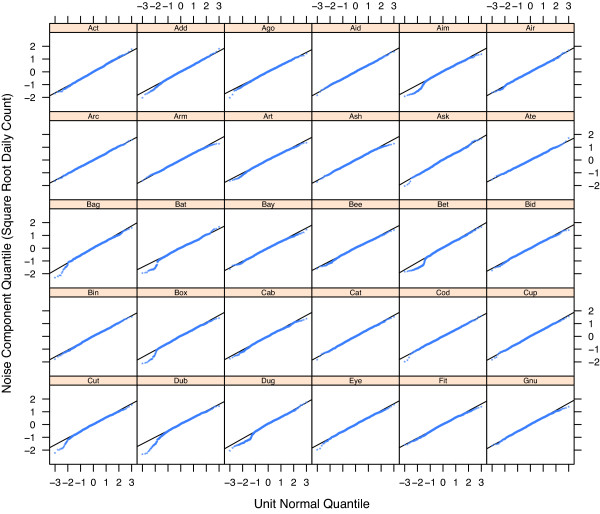
**Normal probability plots for *N*_*t*_**. Normal quantile plots for the noise component, *N*_*t*_, of the square root respiratory counts for 30 EDs. The sample distribution of the noise is well approximated by the normal distribution. This is a result of the square root transformation of the counts.

The four components of variation – *D*_*t*_, *T*_*t*_, *S*_*t*_, and *N*_*t *_– of the STL modeling of the square root respiratory counts, , for the 30 EDs are presented in Figures 3, 4, 5, and 6. To maintain anonymity of the hospitals, names have been replaced by three letter words, and each *T*_*t *_has been altered by subtracting its mean.

The day-of-the-week components, *D*_*t*_, in Figure 3 tend to peak on Sunday or Monday, fall to a minimum Thursday or Friday, and then rise. For some EDs, *D*_*t *_is very high over the weekend but for others is considerably lower.

Figure 4 shows the yearly-seasonal component, *S*_*t*_. The flu season peaks for 2004–2005 and 2007–2008 tend to be larger than those for 2005–2006 and 2006–2007. Many EDs show a double peak for 2006–2007.

Some *T*_*t *_in Figure 5 show an increase and others are nearly constant. We attribute the increase to a growing population using an ED, and no growth to a stable population. Our conclusion is in part based on observing a similar pattern for gastro-intestinal counts.

Diagnostic plots validated the STL modeling. One example, Figure 6, shows normal quantile plots of the *N*_*t*_. The lines on the plots are drawn through the lower and upper quartile points. Because the data of the panels are close to the lines, the normal is a good approximation of the distribution. Plots of the autocorrelation functions of the *N*_*t *_showed the noise component can be modeled as independent random variables. Loess smoothing of the *N*_*t *_against *t *verified that no variation that should have been in *T*_*t *_or *S*_*t *_leaked into the *N*_*t*_. Separate loess plots of the *N*_*t *_for each day-of-the-week verified that all significant day-of-the-week effects were captured by *D*_*t*_.

### Modeling on the Counts Scale

The three systematic components – *T*_*t*_, *S*_*t*_, and *D*_*t *_– are each weighted averages of a large number of observations, so the values are are comparatively stable. The statistical variability in  is chiefly the result of *N*_*t*_, modeled as independent, identically distributed normal random variables with variance . Thus the sample mean of *Y*_*t *_is *λ*_*t *_from Equation 2.

The square root of a Poisson variable whose mean is not too small is approximately normal with a standard deviation of 0.5 [23]. The sample standard deviations  of the 30 noise components are all greater than 0.5, indicating a systematic departure, but are not far from 0.5. The upper quartile of the estimates is 0.58 and the median is 0.54; two estimates reach as high as 0.63 but this is due to a few outliers. The consistency of the values can be seen in Figure 6; the slopes of the lines on the display are nearly the same.

The near normality of *N*_*t *_and the closeness of the  to 0.5 are consistent with the *Y*_*t *_having a distribution that is well approximated by the Poisson with mean *λ*_*t*_. However, in carrying out the detection method, we use  in place of the theoretical value 0.5 to provide a measure of robustness to the Poisson model.

### Outbreak Detection

**Figure 7:**
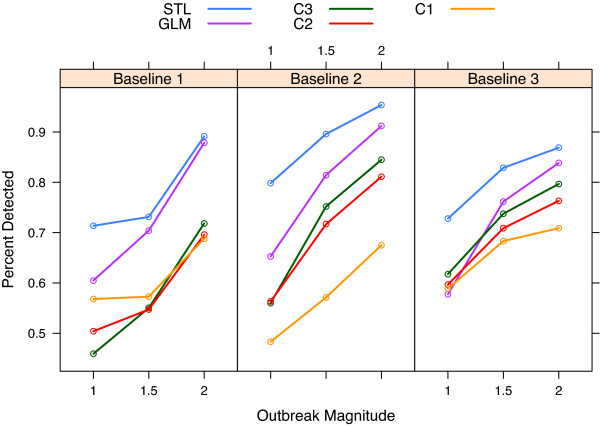
**Outbreak simulation results**. Outbreak detection simulation results. The false positive rate was set empirically for each method and baseline to be 0.03. For each baseline, the STL method detects more than 10% more outbreaks than the other methods at the smallest magnitude.

Table 1 reports the observed sensitivity and average number of days until detection for each baseline, outbreak magnitude, and method. Figure 7 graphs the sensitivity versus magnitude. The days until detection are recorded including the incubation period, and referring to Figure 2, outbreak cases usually do not begin until day 3 or 4. Detection before day 6 or day 7 is before the expected outbreak peak. The C1 method typically has the earliest detection, but the sensitivity is poor. The GLM and STL methods have the best sensitivity, with the STL method clearly ahead in all cases. The STL method detects outbreaks somewhat faster than the GLM method.

**Table 1:** Outbreak detection simulation results.

		Sensitivity					
		
Baseline	Magnitude	C1	C2	C3	GLM	STL	STL(90)
1	1.0	0.57 *(5.44)*	0.50 *(5.50)*	0.46 *(5.48)*	0.60 *(6.58)*	0.71 *(6.44)*	0.66 (6.65)
1	1.5	0.57 *(5.42)*	0.55 *(5.65)*	0.55 *(6.05)*	0.70 *(6.80)*	0.73 *(6.54)*	0.83 (6.57)
1	2.0	0.69 *(5.39)*	0.70 *(5.64)*	0.72 *(6.13)*	0.88 *(6.24)*	0.89 *(6.22)*	0.93 (6.07)
							
2	1.0	0.48 *(5.43)*	0.56 *(5.90)*	0.56 *(5.89)*	0.65 *(6.01)*	0.80 *(6.31)*	0.82 (6.90)
2	1.5	0.57 *(6.03)*	0.72 *(6.68)*	0.75 *(6.81)*	0.81 *(6.72)*	0.90 *(6.63)*	0.89 (5.96)
2	2.0	0.68 *(5.11)*	0.81 *(5.39)*	0.84 *(5.53)*	0.91 *(5.36)*	0.95 *(5.19)*	0.91 (5.93)
							
3	1.0	0.59 *(5.51)*	0.60 *(5.74)*	0.62 *(5.80)*	0.58 *(6.20)*	0.73 *(6.62)*	0.70 (6.74)
3	1.5	0.68 *(6.01)*	0.71 *(6.37)*	0.74 *(6.49)*	0.76 *(7.00)*	0.83 *(6.82)*	0.81 (5.93)
3	2.0	0.71 *(5.47)*	0.76 *(5.82)*	0.80 *(5.87)*	0.84 *(6.21)*	0.87 *(6.00)*	0.87 (5.70)

Outbreak detection simulation results for three baselines and three outbreak magnitudes.

Sensitivity is reported with mean days until detection in parentheses. The false positive rate was set empirically for each method and baseline to be 0.03. The superiority of the sensitivity of the STL method for these scenarios is evident, especially at the smallest outbreak magnitude. The results in the final column, STL(90), are from allowing only a 90 day historical baseline for each outbreak scenario.

At the lowest outbreak magnitude, the STL method outperforms all other methods by a difference of 10% sensitivity. At higher magnitudes, the gap closes, but this is not surprising because all reasonable methods should eventually converge to 100% detection as magnitude increases. Comparisons of sensitivity across baselines within each method and magnitude show quite a bit of variability. This may be due to unique characteristics of each ED, the random outbreak generation, or the fact that the cutoff values were set empirically within each baseline.

The results of running the STL method on the same date range, but using only 90 days of baseline data for each scenario, are shown in final column of Table 1. These numbers are very comparable to the results using all available data. This indicates that the STL detection method is effective when 90 days of data are available.

**Figure 8:**
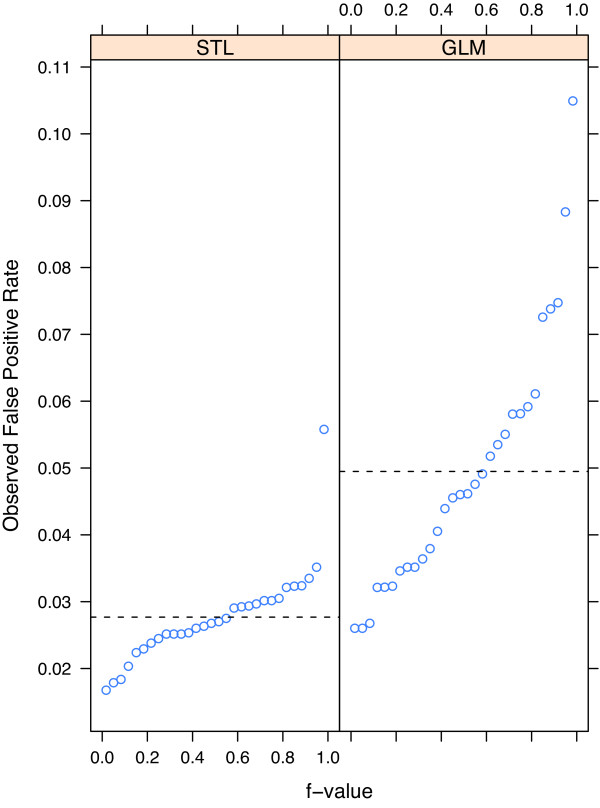
**Observed false positive rates for STL and GLM**. Quantile plots of observed false positive rates for the STL and GLM methods based on a theoretical false positive rate of 0.03, from respiratory counts for each of the 30 EDs. The dashed lines represent the median value for each method.

Figure 8 shows quantile plots of the observed false positive rates from the GLM and STL methods for the historical data with no outbreaks and a theoretical false positive rate of 0.03 for all 30 EDs. The observed rates for the GLM methods deviate from 0.03 by far more than for STL.

To better understand the difference in performance across outbreak methods, we investigated the model fitted values (predicted systematic values) and residuals (data minus the fits) from each method. As we have emphasized, the methods of statistical modeling upon which each detection method is based have a large impact on performance. For the EARS methods, the fitted value for day *t *is the seven-day baseline raw data mean *μ*_*t *_from Equation 4. For the GLM and the STL methods, the fitted value for day *t *is the evaluation of the systematic components at time *t *for a fit through time *t*, just as it would be used in practice for outbreak detection. GLM bases this on data for a number of years; STL bases it on 90 days to a good approximation unless there is substantial long-term trend, which there is not in our Indiana surveillance data.

**Figure 9:**
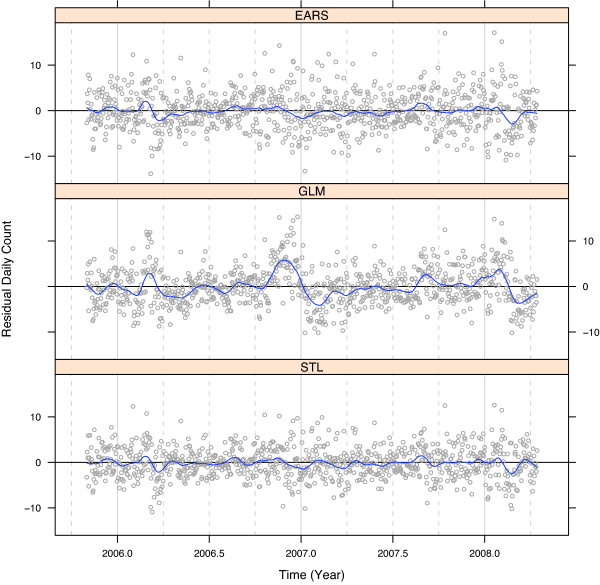
**Residuals for model fits**. Residuals for model fits to daily respiratory counts for one ED. The EARS residuals are the observed count minus the 7 day baseline mean with lag 2. The GLM and STL residuals are obtained from the model predicted values. The smooth curve is the local mean of the residuals.

**Figure 10:**
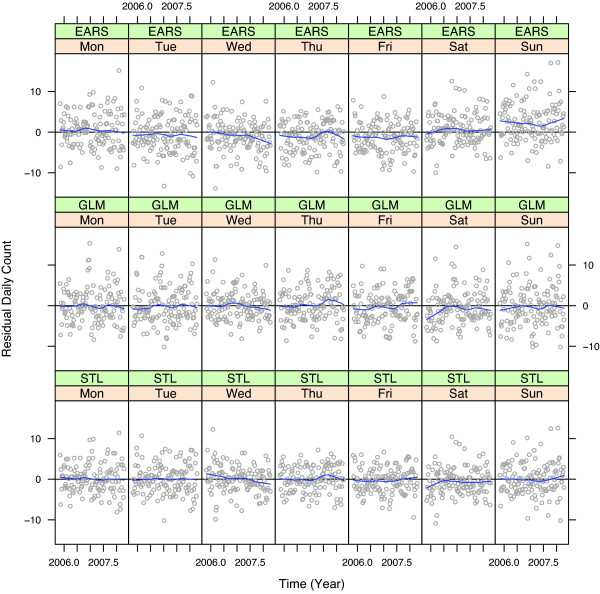
**Residuals by day-of-the-week**. Residuals for model fits to daily respiratory counts for one ED, by day-of-the-week. The smooth curve is the local mean of the residuals.

Figures 9 and 10 are two residual diagnostic plots to check for lack of fit in the EARS, GLM, and STL methods for the daily respiratory counts for one ED. Figure 9 graphs the residuals against *t *for each model; a loess curve shows the local average fluctuations of the residuals around 0. Figure 10 graphs the residuals against week for each day-of-week, also with loess curves. Figure 9 shows substantial lack of fit for GLM, systematic fluctuations from 0. This is due to pooling across years to estimate the yearly seasonal component, which can be quite different from one year to the next. Lack of fit revealed for EARS and STL is minor. Figure 10 shows systematic lack of fit by day of the week for EARS, but not STL and GLM. The reason is simply that EARS does not model day of the week variation.

**Figure 11:**
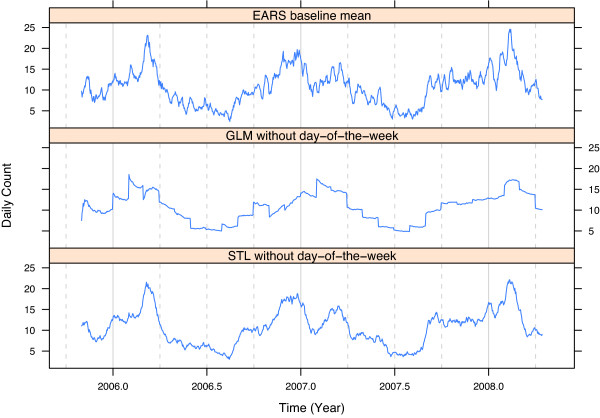
**Fitted components**. Fitted components for daily respiratory counts for one ED. The EARS fitted value at day *t *is the the 7 day baseline mean with lag 2. The GLM and STL fitted values are the predicted values from fitting the models up to day *t *but with the day-of-the-week components removed to make comparison with the variability of the EARS fitted values commensurate.

Figure 11 checks the variability in the EARS, GLM, and STL fitted values for the same ED of the previous two figures. We want the fitted values to be as smooth as possible without introducing lack of fit. For EARS, the fitted values are graphed against *t*. To make comparisons of EARS with GLM and STL commensurate, we graph the GLM and STL fitted values minus their day-of-week components against *t*. The EARS fit is much noisier than that of STL, the result of overfitting. In particular there is a large amount of synoptic scale variation, making it more difficult to detect synoptic-scale outbreaks. GLM is locally quite smooth at most points in time, but has large precipitous jumps at certain time points because the yearly seasonal component is modeled as a step function, constant within months, but changing discontinuously from one month to the next.

## Discussion

For our data, the same smoothing parameters were successfully used across all EDs. We advise those interested in applying these methods to their syndromic data to perform their own parameter selection and model validation. For outbreak detection, our data behaved like Poisson random variables, but this may not be the case in other applications. We advise checking this assumption and if it does not hold, investigating other possibilities such as the over-dispersed Poisson. Another alternative is validating distributional properties of the remainder term and using this term for monitoring.

Although our detection performance results favor the STL method, this does not necessarily mean that it is the best method available. There are certainly other methods with which we could have compared. We chose the EARS methods since they are widely used and can be applied to very short disease time series. Our detection study was limited to one type of outbreak. Further use and study of this method will determine its merit. The visualization capabilities of STL modeling make it a useful tool for visual analytics.

The STL method should not be used for small counts close to and including zero. While they work well with the large counts of the respiratory and gastro-intestinal categories, many other categories such as botulinic have counts that are too small for the square roots to be approximately normally distributed. Future work can investigate employing STL ideas for small counts by replacing the square root normal distribution and local least-squares fitting with the Poisson distribution and local Poisson maximum likelihood fitting.

## Conclusion

The STL decomposition methods presented here effectively model chief complaint counts for syndromic surveillance without significant lack of fit or undue noise, and lead to a synoptic time-scale disease outbreak detection method that in our testing scenarios performs better than other methods to which is it compared – EARS C1, C2, and C3 methods [4], as well as a Poisson GLM modeling method [24]. The methods can be used for disease time series as short as 90 days, which is important because many surveillance systems have started only recently and have a limited number of observations. Visualization of the components of variation – inter-annual, yearly-seasonal, day-of-the-week, and random-error – provides much insight into the properties of disease time series. We recommend the methods be considered by those who manage public health surveillance systems.

## Competing interests

The authors declare that they have no competing interests.

## Authors' contributions

DSE, WSC, and SJG designed the research project, obtained funding, and recruited other members. RPH, DA, and WSC developed and implemented the statistical methods and wrote the first draft of the manuscript. RPH developed the disease outbreak detection method and carried out the simulations. RPH, DA, WSC, and RM conducted the literature review. AA, MY, MO, and SJG designed the data collection, the database schema, and the form of the continuous data feed. All authors participated in the review of the manuscript and approved it.

## Pre-publication history

The pre-publication history for this paper can be accessed here:

http://www.biomedcentral.com/1472-6947/9/21/prepub

## Supplementary Material

Additional file 1 **R code and documentation**. contains R source code, examples, data, and documentation for carrying out the STL procedure. Click here for file
