# Supplementary material for: Syndromic surveillance: STL for modeling, visualizing, and monitoring disease counts
Source: BMC Med Inform Decis Mak. 2009 Apr 21;9:21. doi: 10.1186/1472-6947-9-21 (PMC2680402; doi:10.1186/1472-6947-9-21)
Supplement: Additional file 1 — R code and documentation. contains R source code, examples, data, and documentation for carrying out the STL procedure. [file 1472-6947-9-21-S1.zip › documentation.pdf]

`bmcstl-package` *STL for Syndromic Surveillance*

Description

STL decomposition for modeling and visualization of disease count data and detection of disease outbreaks.

Details

Package:	bmcstl
Version:	0.1-1
License:	GPL (version 2 or later)

Index:

This purpose of this package is to illustrate how to implement the methods presented in this paper in R. All routines necessary for STL modeling as described in this paper are available in the base distribution of R. The functions provided come with some limited flexibility. For example, a vector 1, ..., n is used as the time variable and the day-of-the-week is coded as a generic 1, ..., 7. We advise those interested in customizing the procedure to their own needs to view the source of the functions to see what they do and use this as a starting point.

For some example code, see the attached file "example.R" or see `bmcstl` along with the other function definitions below.

This was originally put together as a package, but due to the difficulty some might have installing the package source and due to the simplicity of the package, instead we are supplying all of the code in one R source file "code.R", the data in another file "respsim.R", and example code in another file "example.R", along with this documentation file.

To run the code, open an R session and change your working directory to the directory that these files reside in. This can be done with the `setwd()` command or through the GUI on Mac or Windows. Then follow the code in example.R.

Author(s)

Ryan Hafen <rhafen@purdue.edu>

Maintainer: Ryan Hafen <rhafen@purdue.edu>

References

R. B. Cleveland, W. S. Cleveland, J.E. McRae, and I. Terpenning (1990) STL: A Seasonal-Trend Decomposition Procedure Based on Loess. *Journal of Official Statistics*, 6, 3-73.

W.S. Cleveland, E. Grosse and W.M. Shyu (1992) Local regression models. Chapter 8 of *Statistical Models in S* eds J.M. Chambers and T.J. Hastie, Wadsworth & Brooks/Cole.

See Also

bmcstl, plot.bmcstl, dowplot, plot.seasonal, plot.trend

---

bmcstl *STL decomposition*

Description

Obtains the STL decomposition as described in the paper

Usage

```
bmcstl(counts, s.deg = 0, s.span = "periodic", t.deg = 1, t.span =
39, ia.deg = 1, ia.span = 1000, ys.deg = 2, ys.span = 91, trnsfrm = sqrt,
blend = FALSE, blend.prop = 1, blend.n = 50, blend.ys.span =
as.integer(ys.span/2))
```

Arguments

counts	a vector of daily counts values, untransformed.
s.deg	seasonal degree of smoothing for the day-of-the-week component extraction.
s.span	seasonal local bandwidth (in days) for the day-of-the-week component extraction. Also, s.span="periodic" (default) is an option, which results in the seasonal smoothing being an average.
t.deg	trend degree of smoothing for the day-of-the-week component extraction.
t.span	trend local bandwidth (in days) for the day-of-the-week component extraction.
ia.deg	degree of smoothing for the inter-annual component.
ia.span	local bandwidth (in days) for the inter-annual component.
ys.deg	degree of smoothing for the yearly-seasonal component.
ys.span	local bandwidth (in days) for the yearly-seasonal component.
trnsfrm	transformation function to use. It is recommended to use the default sqrt.
blend	logical. Whether to blend to degree 0 at the extremes.
blend.prop	the proportion of blending upon reaching the endpoint. Numeric value between 0 and 1. Not used if blend=FALSE.
blend.n	how many days before the end (and after the beginning) to start the linear blending to degree 0. Not used if blend=FALSE.
blend.ys.span	the local bandwidth for the degree 0 smooth. Not used if blend=FALSE.

Details

The default parameters are those used in the paper.

This function uses R's `stl()` function to extract the day-of-the-week component. Then it subtracts this from the daily counts and smooths the result using `loess()` to obtain the inter-annual component. Then the yearly-seasonal component is extracted from the counts minus the day-of-the-week component minus the inter-annual component.

Value

A list of class "bmcstl"

`bmc.stl`	object of class "stl". The result of the day-of-the-week stl decomposition.
`out`	data frame of the components: `n`=daily counts, `t`=time vector, `c_raw`=the transformed counts, `c_dow`=day-of-the-week component, `c_ys`=yearly-seasonal component, `c_ia`=inter-annual component, `c_n`=noise component.

Author(s)

Ryan Hafen

References

R. B. Cleveland, W. S. Cleveland, J.E. McRae, and I. Terpenning (1990) STL: A Seasonal-Trend Decomposition Procedure Based on Loess. *Journal of Official Statistics*, 6, 3-73.

W.S. Cleveland, E. Grosse and W.M. Shyu (1992) Local regression models. Chapter 8 of *Statistical Models in S* eds J.M. Chambers and T.J. Hastie, Wadsworth & Brooks/Cole.

See Also

`plot.bmcstl`, `dowplot`, `plot.seasonal`, `plot.trend`

Examples

```
library(bmcstl)

# synthetic data set
data(respsim)

# obtain the decomposition using the default parameters (as in the paper)
decomp1 <- bmcstl(respsim)

# plot the decomposition
plot(decomp1)
# view the stl decompostion used for extracting day-of-the-week
plot(decomp1$dow.stl)
# view the day-of-the-week component by day-of-week
dowplot(decomp1)

### diagnostics
```

```
# first, check the day-of-the-week component stl decomposition
# check to see if the periodic day-of-the-week is a good assumption
plot.seasonal(decomp1)
plot.trend(decomp1)

# now look at the noise component
# now check the residuals of the fit
dd <- noise(decomp1)

# normal probability plot
qqnorm(dd)
qqline(dd)

# autocorrelation plot
plot(acf(dd))

# plot of residuals with loess smooth
plot(dd, col="darkgray")
dd.loess <- loess(dd ~ c(1:length(dd)), span=60/length(dd))
lines(predict(dd.loess), col="red", lwd=2)

# perhaps try a smaller yearly-seasonal window
decomp2 <- bmcstl(respsim, ys.span=61)
plot(decomp2)

dd2 <- noise(decomp2)

qqnorm(dd2)
qqline(dd2)
plot(acf(dd2))

plot(dd2, col="darkgray")
dd2.loess <- loess(dd2 ~ c(1:length(dd2)), span=60/length(dd2))
lines(predict(dd2.loess), col="red", lwd=2)
```

`dowplot` *Day-of-the-week component plot*

Description

Plots the fitted day-of-the-week component vs. time, as discussed in Cleveland et. al. 1990. With `s.span="periodic"`, this will just be a straight line for each day, but for other parameter choices, this will show how the component changes over time for each day.

Usage

```
dowplot(x, layout = c(7, 1), xlab = "Time", ylab = "", ...)
```

Arguments

`x`	object of class "bmcstl" obtained from `bmcstl`.
`layout`	Describe `layout` here
`xlab`	x-axis label for the plot.
`ylab`	y-axis label for the plot.
`...`	other graphics parameters to be passed to xyplot.

Value

object of class "trellis"

Author(s)

Ryan Hafen

References

R. B. Cleveland, W. S. Cleveland, J.E. McRae, and I. Terpenning (1990) STL: A Seasonal-Trend Decomposition Procedure Based on Loess. *Journal of Official Statistics*, 6, 3-73.

See Also

`bmcstl`, `plot.bmcstl`, `plot.seasonal`, `plot.trend`

`plot.bmcstl` *STL Decomposition Plot*

Description

Plots each component of variation from the STL decomposition. By default, the y-axis has the same scale for each panel.

Usage

```
plot.bmcstl(x, layout = c(1, 5), main = "STL Decomposition", ...)
```

Arguments

`x`	object of class "bmcstl" obtained from `bmcstl`.
`layout`	layout of the lattice xyplot.
`main`	title of the plot.
`...`	other graphics parameters to be passed to xyplot.

Value

object of class "trellis"

Author(s)

Ryan Hafen

See Also

bmcstl, bmcstl, plot.seasonal, plot.trend

---

plot.seasonal — *Seasonal diagnostic plot for day-of-the-week component in STL decomposition*

Description

Plots the residuals from the day-of-the-week STL decomposition along with the fitted values, as discussed in Cleveland et. al. 1990. Used to assess the `s.span` and `s.deg` parameter choices.

Usage

```
plot.seasonal(x, par.settings = list(superpose.symbol = list(col =
"darkgray"), superpose.line = list(col = "blue", lwd = 2)), ...)
```

Arguments

`x`	object of class "bmcstl" obtained from `bmcstl`.
`par.settings`	graphics parameter settings for lattice.
`...`	other graphics parameters to be passed to xyplot.

Value

object of class "trellis"

Author(s)

Ryan Hafen

References

R. B. Cleveland, W. S. Cleveland, J.E. McRae, and I. Terpenning (1990) STL: A Seasonal-Trend Decomposition Procedure Based on Loess. *Journal of Official Statistics*, 6, 3-73.

See Also

bmcstl, plot.bmcstl, dowplot, plot.trend

plot.trend — *Seasonal diagnostic plot for day-of-the-week component in STL decomposition*

Description

Plots the raw data with the fitted trend component used to extract the day-of-the-week component along with the residuals, as discussed in Cleveland et. al. 1990. Used to assess the `t.span` and `t.deg` parameter choices. Note the the trend here is not the inter-annual or yearly-seasonal. This is the trend in the day-of-the-week extraction.

Usage

```
plot.trend(x, xlab = "Time", ylab = "", par.settings = list(superpose.symbol
= list(col = "darkgray"), superpose.line = list(col = "blue", lwd = 2)),
...)
```

Arguments

`x`	object of class "bmcstl" obtained from `bmcstl`.
`xlab`	x-axis label for the plot.
`ylab`	y-axis label for the plot.
`par.settings`	graphics parameter settings for lattice.
`...`	other graphics parameters to be passed to xyplot.

Value

object of class "trellis"

Author(s)

Ryan Hafen

References

R. B. Cleveland, W. S. Cleveland, J.E. McRae, and I. Terpenning (1990) STL: A Seasonal-Trend Decomposition Procedure Based on Loess. *Journal of Official Statistics*, 6, 3-73.

See Also

`bmcstl`, `plot.bmcstl`, `dowplot`, `plot.seasonal`
